# The Advancement of the Science of Healing

**Published:** 1914-12-26

**Authors:** 


### THE ADVANCEMENT OF THE SCIENCE OF HEALING.*

### By the Right Hon. the Viscount Midleton.

I have been asked to say a word or two in regard to the work of this League of Mercy; but I think you will agree that what has already been said by the experienced officials who have addressed you covers all the ordinary work of the League. Last year you had the opportunity of hearing a most attractive and impressive address from Father Vaughan, who was able to speak, with a freedom which I could not attempt to emulate, on the duties which we owe, one class towards another, in this country. And perhaps I may be allowed to say one word as regards the work of this League from the point of view with which you are at present very much in touch, and of which I have some personal knowledge—namely, the extent to which this League, and some other organisations, have advanced the whole science of healing and of hospital work, and have therefore contributed, perhaps more than any other body in this country, to the mitigation of the suffering caused by this present war. We are all proud of the immense personal energy which people of all classes in this country have shown in the last few months in endeavouring, if not personally engaged in the war, to do their share, whatever it may be, either towards alleviating suffering or preventing hardship, or, in the case of our Allies, cheering the victims by hospitality or even assisting in the preparation of troops. But I am afraid that, like all our military preparations, this work has been done in a hurry, on the spur of the moment, and not in the organised fashion in which we might have set to work if we had had notice.

An Organised System of Personal Service.

What I venture to claim for this League is, that in their work in assisting with the equipment of hospitals all over the home counties and London, they have pursued it as a policy, as an organised system, for many years, involving heavy demands on the individual and great claims on personal service, but setting a perfectly matchless example. I remember a speech which Lord Milner made on the rather debateable subject of National Service about two years ago. He said everybody was prepared to work in an emergency: the difficulty was that people would not make sacrifices before the emergency arose; and he cited in support of his argument a recent speech made by a member of Parliament, sitting on which side of the House it would not be prudent for me at this moment to say, in which that member said, "Why make all these preparations? If an enemy landed a million bayonets would flash from their scabbards." To this Lord Milner added, "It would be very easy if the bayonets existed in their scabbards." This reminded me of a remark made by an old lady as she was leaving church after hearing the chapter read which ended, "There shall be weeping and gnashing of teeth." She said, "Let them as has teeth gnash 'em; I can't." (Laughter.)

And I really venture to say that we here who have belonged to the League of Mercy for some years, who have done our share, as far as we can, in carrying out the great policy which the late King inaugurated on Hospital Sunday, and which he pressed far in regard to the reform of medical administration in the Army, have done something towards the rapid advancement of the science of healing. I was thinking, as I looked round at the pictures of successive monarchs on this wall, if we could induce them all to come down from their canvases and join us in a discussion as to what was the most remarkable progress in this country from the time of King Henry VIII. to that of His late Majesty, they would all be forced to admit it was not the extension of territory, or the reform of customs, or the advance of comforts of all classes, or even the great advancement of the law. All those things have come gradually, dating from centuries back, not by any specially rapid movement in the last few years. But I think they would, one and all, agree—and some of them were the victims of the kind of science practised by the medical profession—that the one subject in England which, more than any other, has advanced in the last fifty years with great and rapid strides, is the whole science of medicine—doctoring, surgery, and nursing.

It is not difficult to understand, therefore, why any work connected with hospitals is not always the most peaceful; there must be differences of opinion when there has been such a rapid advance in science. We can remember what nurses were when Dickens drew his famous picture of Mrs. Gamp; we have learned what they were from our parents forty or fifty years ago; we know how far removed they were from the admirable ladies—keen, hard-working, always to the fore—who now attend us in moments of illness. We know, also, what hospitals were fifty, seventy, or eighty years ago. Perhaps the greatest social advance in this country is that the poor man who finds his way into a hospital now is sure to be quite as well looked after and be as carefully tended as the richest man can be in his own house. And, of course, the change in doctors has been very great also. I am not at all certain that there was not a good deal to be said for the rough-and-ready practitioners who in old days formed the majority of the medical profession. I remember a doctor I knew in my early days, who knew me from the earliest possible moment of my life, and I said to him one day, "To what do you attribute the large fortune that you have made in your big practice?" He was a man who always thought you wanted "keeping up." He said, "I always kept up my patients, and I got a tremendous practice among the Guards' young men when they found I was in the habit of ordering port for the gout." (Laughter.) And I remember another rough-and-ready

* Address delivered at the annual meeting of Presidents of the League of Mercy, December 17, 1914.

doctor, who said, "I have not got the scientific attainments of some of my brethren, but I have done pretty well. I go on the principle that most of my rich patients eat too much; all my poor patients eat too little; and whatever else I do for them I follow strictly the Scriptural maxim; I fill the hungry with good things, and the rich I send empty away." (Laughter.) Those doctrines are good general ones to go upon. But we must not be surprised if between that system and the highly organised system of the present day there is some hiatus. I remember a friend who said to me, "I do not believe in the operation for appendicitis, but it was performed on me, and I did not think it had done me any good. When I got ill again another surgeon seized hold of me and said, 'Let me take out your appendix.' I said 'You can't do that, I have had it done already.' 'Ah,' he said, 'it was not done properly,' and he had another go." (Laughter.)

### A Democratic Work.

There will be and must be difficulties in connection with the highly organised system of medical work under which we live. Still, the main fact remains, that all these advances need considerable funds to keep them going. The hospitals of London have been forced to be beggars from time immemorial, and nothing has been so instrumental in relieving the hourly anxieties of hospitals as King Edward's hospital scheme, and the help which has been given to it by the League of Mercy. And if I had Father Bernard Vaughan here I should like to remark to him that just as we have every day tributes from our forces in the field as to the manner in which our officers have come out in this campaign and contributed to the gallantry of their comrades, and shown the unity which exists between our troops, so I think in this more civil and peaceful work in which we are engaged there is nothing more remarkable in the Treasurer's statement than the degree to which the poorest classes have been brought into touch with that work, carried on by those we see around us, and have contributed their share. A League which at the same time that it benefits a noble profession also successfully unites various classes of the community and obtains consistent support, is one which requires very few words to commend it to your notice. We may congratulate ourselves that in this extremely difficult year, when so many attractive programmes have been put before us, appealing to all that is best and most patriotic in us, we have so good a report as we have on this occasion. We may, I say, congratulate ourselves on this, and also on the fact that her Royal Highness, having been so kind as to attend, has received such excellent support on this occasion. (Cheers.)

